# A rapid multiplex PCR assay for presumptive species identification of rhinoceros horns and its implementation in Vietnam

**DOI:** 10.1371/journal.pone.0198565

**Published:** 2018-06-14

**Authors:** Kyle M. Ewart, Greta J. Frankham, Ross McEwing, Dang Tat The, Carolyn J. Hogg, Claire Wade, Nathan Lo, Rebecca N. Johnson

**Affiliations:** 1 Australian Centre for Wildlife Genomics, Australian Museum Research Institute, Sydney, New South Wales, Australia; 2 The University of Sydney, School of Life and Environmental Sciences, Faculty of Science, Sydney, New South Wales, Australia; 3 TRACE Wildlife Forensics Network, Edinburgh, Scotland; 4 Institute of Ecology and Biological Resources, Hanoi, Vietnam; 5 Zoo and Aquarium Association Australasia, Mosman, New South Wales, Australia; University of Helsinki, FINLAND

## Abstract

Rhinoceros (rhinos) have suffered a dramatic increase in poaching over the past decade due to the growing demand for rhino horn products in Asia. One way to reverse this trend is to enhance enforcement and intelligence gathering tools used for species identification of horns, in particular making them fast, inexpensive and accurate. Traditionally, species identification tests are based on DNA sequence data, which, depending on laboratory resources, can be either time or cost prohibitive. This study presents a rapid rhino species identification test, utilizing species-specific primers within the cytochrome b gene multiplexed in a single reaction, with a presumptive species identification based on the length of the resultant amplicon. This multiplex PCR assay can provide a presumptive species identification result in less than 24 hours. Sequence-based definitive testing can be conducted if/when required (e.g. court purposes). This work also presents an actual casework scenario in which the presumptive test was successfully utlitised, in concert with sequence-based definitive testing. The test was carried out on seized suspected rhino horns tested at the Institute of Ecology and Biological Resources, the CITES mandated laboratory in Vietnam, a country that is known to be a major source of demand for rhino horns. This test represents the basis for which future ‘rapid species identification tests’ can be trialed.

## 1. Introduction

The illegal wildlife trade is a multi-billion dollar transnational industry that constitutes one of the top five forms of black market activities [1]. Wildlife forensics is an important tool for wildlife law enforcement and managing wildlife trade activities. DNA-based wildlife forensic science is an evolving discipline which combines techniques utilized by conservation genetics and human forensic science, and their application in the legal system [2]. Technological advancements and the copious genomic sequence data now available offer the potential to improve wildlife forensic techniques and hence enforcement [3]. However, countries with limited financial resources and/or infrastructure capacity may have difficulties to consistently carry out DNA-based forensic testing, especially when faced with numerous large seizures. Additionally, in some juristictions there may be strict time constraints on intelligence gathering or statutes of limitations. The development of rapid and cost-effective wildlife forensics techniques is therefore vital for laboratories that are subject to such constraints, so that they may increase conviction rates and improve enforcement outcomes.

Rhinoceros (rhino) numbers have been devastated over the past century, in particular the last decade, as the inflated price of rhino horn has driven an increase in poaching and the illegal trafficking of their horns [1, 4]. Currently there are five extant rhino species; two African species which include *Ceratotherium simum* (white rhino) and *Diceros bicornis* (black rhino), and three Asian species which include *Rhinoceros unicornis* (Indian rhino), *Rhinoceros sondaicus* (Javan rhino) and *Dicerorhinus sumatrensis* (Sumatran rhino). Trade in rhino horns is regulated by the Convention on International Trade in Endangered Species of Wild Fauna and Flora (CITES) which came into force in 1975 [5], and is implemented via national laws of member countries or parties.

In the case of rhinos, successful conservation requires a focus on enhancing the coordination of detection and enforcement of these highly traded species [2, 4]. DNA-based wildlife forensics is one such measure that can be utilized in rhino horn trafficking investigations to confirm the presence of rhino horn and to identify the rhino species of origin. Due to the high price of horn, there has reportedly been many fake/substitute rhino horn products circulating the market, usually made from water buffalo horn, but these can also be made from other keratins, caseins, resins, wood, hair or plastics [4, 6]. Therefore, the first aim in an investigation is to determine whether the seized product is real rhino horn [7]; and secondly, if the seized product is indeed rhino horn, to determine the species from which it originated.

DNA-based individualization of seized horns (or horn derivatives) from two of the species is well established using the rhino DNA indexing system (RhODIS) [8, 9]. RhODIS, a South African (University of Pretoria) based system, is a database containing black and white rhino microsatellite genotypes, designed to capture data from live animals, poached animals and seized items which may aid prosecutions and provide valuable intelligence in regards to tracking horn trade networks globally (for example, linking a seized horn to the individual carcass from which it was poached) [9]. However, for seizures that occur outside South Africa, there are animal health requirements that must be considered before samples can be sent to the University of Pretoria for testing [10]. The threat of foot and mouth disease complicates the import of *Bovidae* species (e.g. water buffalo and domestic cattle) into South Africa. Therefore, species identification testing of seized horn samples (which are potentially a *Bovidae* species) will streamline the transfer of white and black rhino horn samples for subsequent profiling at the RhODIS laboratory [7, 9].

DNA-based species identification protocols are often based on sequence differences of the cytochrome b (cyt b) gene [11, 12]. Previous work validated a protocol that utilizes a 230 base pair (bp) region within cyt b and demonstrated that this gene region is appropriate to use for species identification of rhino horns [6]. However, in some laboratories, without in-house sequencing facilities and/or budgetary constraints, sequence-based methods can be expensive and time consuming. For example, at the Institute of Ecology and Biological Resources (IEBR) in Vietnam, sequencing of PCR products can take over two weeks.

The aims of this study were two-fold:

1. To develop a rapid and low rhino horn species identification protocol based on a multiplex PCR assay, whereby a presumptive species identification for white rhino, black rhino, and Indian rhino can be undertaken based on the size of the resultant amplicons. The purpose of the test is to triage seizure samples and feed these rapid ‘presumptive’ results into trafficking investigations, and to provide an inexpensive platform to identify the species of seized horns (or horn derivatives) prior to sending them to South Africa to become part of RhODIS. This test would complement more time-consuming sequence based ‘definitive’ species identification tests which can be carried out in due course on seized items that require forensic court evidence if/when required.
2. To field test these methods on a real rhino horn seizure at Vietnam’s IEBR, a CITES mandated ‘frontline’ wildlife forensics laboratory, in concert with sequence-based definitive testing. It is hoped successful implementation of this test at IEBR will improve the enforcement and conviction rate of rhino horn trafficking crimes in Vietnam, a country known to be a major source of demand for rhino horns [4].

## 2. Methods

### 2.1 Multiplex PCR assay development

The development of the multiplex PCR assay was undertaken at the Australian Centre for Wildlife Genomics (ACWG), an ISO 17025 accredited laboratory, at the Australian Museum Research Institute (AMRI).

#### 2.1.1 Samples and DNA extraction

The same Australian Museum registered samples from all five rhino species, water buffalo and human used in [6] (excluding the international testing samples) were used in this study for primer design and testing, and multiplex optimization. The Australian Museum Animal Care and Ethics Committee approved the methods used to collect samples from living (captive) individuals for this project, under the Animal Research Authority Project number 14–05. We trialed the assay using 10 known rhino samples and field tested the assay using 60 unknown horn samples from a real seizure (S1 Table). Different subsampling and DNA extraction protocols were used depending on the sample type (i.e. tissue, blood, hair, bone or horn) following the methods described in [6].

#### 2.1.2 Multiplex PCR assay design

The cyt b sequence database established in [6] was utilized for primer design. Fixed SNPs within each of the species (i.e. a nucleotide that occurs in one species but not in other species) were identified in the black rhino, white rhino and Indian rhino sequences using MEGA version 6.06 [13]. Species-specific primers for these three species were designed across regions incorporating these informative SNPs (see Table 1 for primer details). Primers were designed to completely match their target species, and contain at least two mismatches (mostly towards the 3' end of the primer) with non-target rhino species (i.e. Javan rhino and Sumatran rhino), water buffalo (a commonly substituted horn) and human (a likely contaminant). Primers were designed by eye based on sequence alignments in MEGA version 6.06 [13] and checked for melting temperatures, potential hairpins and primer dimer interactions using OLIGO 7 primer analysis software [14]. These cyt b species-specific primers were incorporated into the design of a multiplex PCR. This assay generates amplicons of three different lengths that are unique to each of the three target rhino species (black rhino, white rhino and Indian rhino).

**Table 1:** Cytochrome b markers and their corresponding primers.

Genetic marker for:	Primer name:	Primer sequence (5’–3’):	Annealing temperature (°C):	Amplicon length (bp):	Reference:
*Diceros bicornis* (black rhino)	Rh_BR_FWD (forward)	AATCTGCCTAATCCTACAAATC	60	222	This study
	Rh_BR_REV (reverse)	GGTTTCTAGGAAGGTGTAGG			
*Ceratotherium simum* (white rhino)	Rh_WR_FWD (forward)	CCACTCATTCATCGATCTGC	60	266	This study
	Rh_WR_REV (reverse)	TAATAGATACCGCGTCCTAC			
*Rhinoceros unicornis* (Indian rhino)	Rh_IR_FWD (forward)	TCTCACCCACTAGTTAAAATCA	60	310	This study
	Rh_IR_REV (reverse)	AGGAAGGTGTAAGATCCATAG			
All rhino species	RID_FWD (forward)	AACATCCGTAAATCYCACCCA	55	230	[6]
	RID_REV (reverse)	GGCAGATRAARAATATGGATGCT			
All rhino species	Mac_FWD (forward	CAYTATACACCAGACACAACAAC	55	182	This study
	Mac_REV (reverse)	TGAAYGCDGTGGCTATTAGRG			

This multiplex PCR was performed in 25 μl of reaction mixture containing 1x Bioline MyTaq Red Reagent Buffer, 80 nM of Rh_WR_FWD primer and Rh_WR_REV primer, 56 nM of Rh_BR_FWD primer and Rh_BR_REV primer, 28 nM of Rh_IR_FWD primer and Rh_IR_REV primer, 1 unit of Bioline MyTaq DNA Polymerase and <2 ng of genomic DNA (using significantly more than ~2 ng of DNA may cause difficulties in visualizing the length of the amplicon on an agarose gel, unless the resultant PCR product is diluted). The PCR conditions in this protocol were as follows: an initial denaturation at 94°C for 3 minutes, followed by 38 cycles of 94 °C for 20 seconds, 59°C for 45 seconds and 72°C for 40 seconds, and an end extension period at 72°C for 5 minutes. The PCR protocol was optimized for low-template horn samples, hence 38 cycles were used; however, using 30 cycles is more appropriate for higher quantity DNA extracts (e.g. DNA from tissue).

Javan rhino, Sumatran rhino, water buffalo and human samples were used to test the specificity of the assay. The assay was always complemented by a sequence based protocol, using RID_FWD/RID_REV primers (Table 1), to confirm the presumptive result produced by the multiplex PCR assay and to exclude a false negative result being reported via a ‘non-amplification’ or no result.

#### 2.1.3 Multiplex PCR trial

The assay was trialed on 10 known samples from three black rhinos, three white rhinos and four Indian rhinos (S1 Table). Different sample types were used to test the utility of the assay, including rhino horn, bone, hair, blood and tissue. The presence and length of multiplex PCR products were assessed using 2% TAE agarose gel electrophoresis stained with GelRed (Biotium), with the inclusion of a 100 bp DNA ladder (ThermoFisher). Positive control samples from known white rhino (M.47191), black rhino (M.46281) and Indian rhino (M.39431) from the Australian Museum Mammal Collection were run alongside the unknown sample on the agarose gel to provide an effective means to visualize the amplicon size and hence identify the species of the unknown samples. Representative positive amplifications were purified using ExoSap-IT and Sanger sequenced at the Australian Genome Research Facility (Sydney, Australia) to ensure target species have indeed been amplified and no cross species amplification had occurred. Sequences were compared to those generated from the available reference specimens and sequences available on GenBank to confirm correct species identification.

### 2.2 Implementation and field test at IEBR

The multiplex PCR assay was field tested and implemented at IEBR in Hanoi, Vietnam. The methods undertaken during the field test integrated the previously described methods (see Section 2.1) modified within the capabilities of IEBR’s existing infrastructure and procedures.

#### 2.2.1 IEBR samples and extraction method

Sixty horn fragments of unknown species origin (each representing a different individual horn) from past seizures held by IEBR were tested in their laboratory (S1 Table). The subsampling and extraction method at IEBR was similar to that carried out in [6], with some minor modifications made due to differing laboratory equipment/reagents. Samples too small to drill were cut to ~2 mm^3^ with a pair of scissors. Approximately half of the 60 horn fragments were homogenized with a Qiagen TissueRuptor homogenizer, and 20 μL of dithiothreitol (DTT) (~1 M) was added to all of the samples during the lysis step. Formal experiments testing the significance of drilling and homogenizing the horn were not undertaken in this study.

#### 2.2.2 IEBR multiplex PCR assay methods

The multiplex PCR assay was conducted on all 60 horn samples using reagents available at IEBR: amplification was performed in 8.82 μl of reaction mixture containing 7 μl of QuantiTect SYBR Green PCR Master Mix (QIAGEN), 0.2 μl of Rh_WR_FWD primer and Rh_WR_REV primer (10 μM of each), 0.14 μl of Rh_BR_FWD primer and Rh_BR_REV primer (10 μM of each), 0.07 μl of Rh_IR_FWD primer and Rh_IR_REV primer (10 μM of each) (see Table 1 for primer details) and 1 μl of genomic DNA (of unknown concentration; no quantification equipment was available). Amplicons were assessed using 2% TAE agarose gel electrophoresis stained with ethidium bromide. The multiplex PCR assay species identification results were confirmed using a sequence based species identification method on the same DNA samples using ‘universal rhino primers’ developed by the ACWG (RID_FWD/RID_REV and Mac_FWD/Mac_REV; see Table 1).

## 3. Results

**Fig 1:**
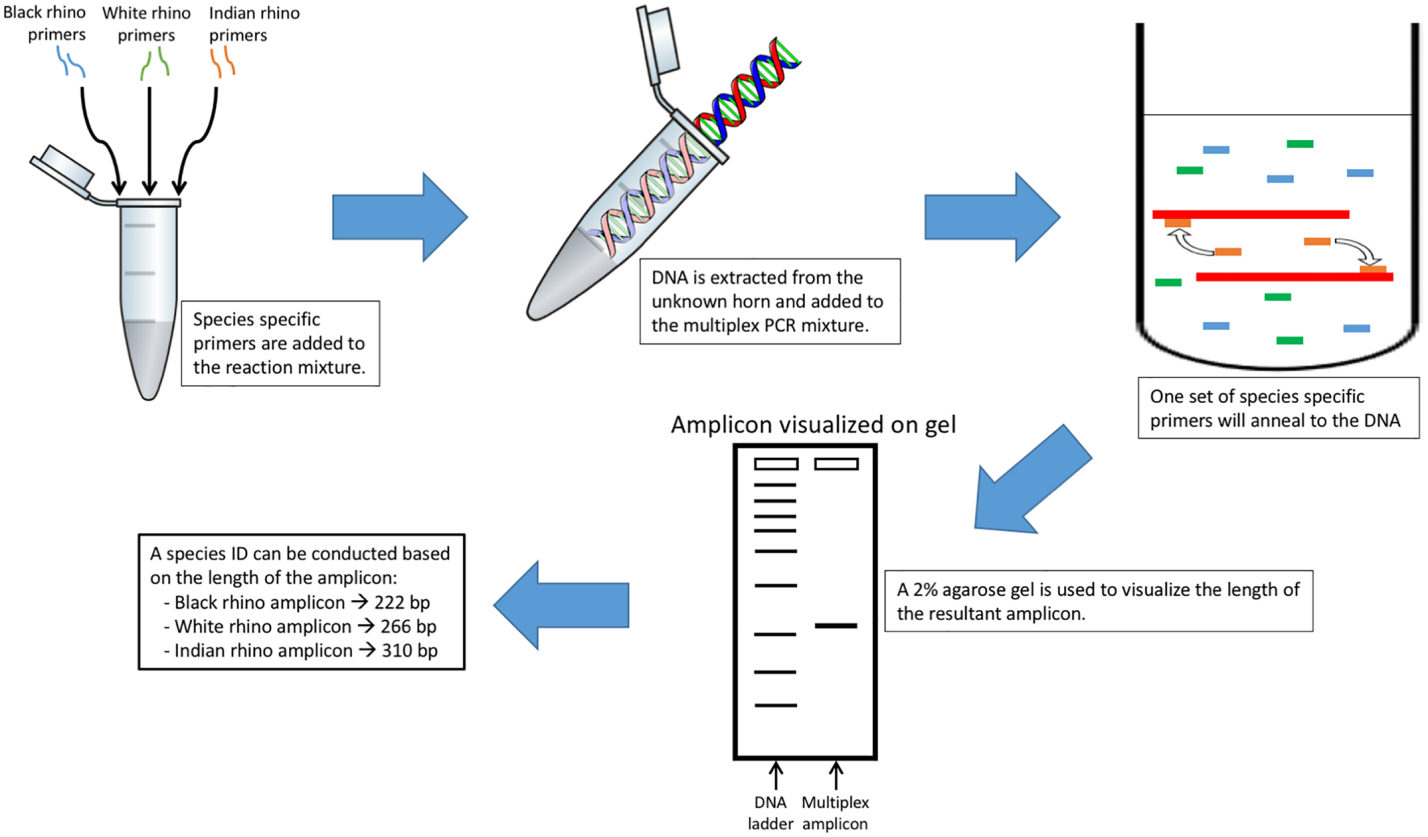
Multiplex PCR assay protocol: Identifying an unknown rhino specimen.

This newly designed multiplex PCR assay protocol, illustrated in Fig 1, is able to provide a presumptive species identification for black rhino, white rhino and Indian rhino in less than 24 hours on the basis of an amplicon length that is species-specific.

### 3.1 Assay development and laboratory trial carried out at the ACWG

When testing the multiplex PCR on non-target species (i.e. the Javan rhino, Sumatran rhino, water buffalo and human), no amplicons were detected.

The multiplex PCR assay successfully identified 100% (10 out of 10) of the known samples tested during its development stage (S1 Table). Successful amplicons were sequenced and determined to be of the target species when compared to reference sequences.

### 3.2 Field test carried out at IBER

**Fig 2:**
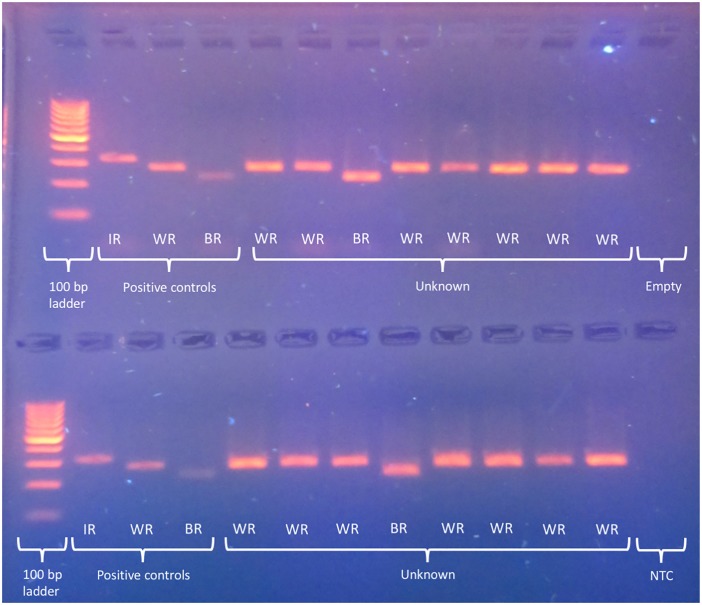
A 2% agarose gel run at IEBR showing the amplicons for 16 unknown horn samples using the multiplex PCR assay. The positive controls are known samples from Indian rhino (M.39431.001; 310 bp amplicon) white rhino (M.47191.001; 266 bp amplicon) and black rhino (M.46281.001; 222 bp amplicon). Based on this gel, all of the unknown samples are from white rhino (14 out of 16) and black rhino (2 out of 16) horn. NTC = no template control; IR = Indian rhino amplicon; WR = white rhino amplicon; BR = black rhino amplicon.

In the field test, 83% (50 out of 60) of the unknown horn samples were successfully identified with the multiplex PCR assay (S1 Table). All of the multiplex PCR assay identifications were confirmed by their DNA sequence generated using the ‘universal rhino primers’ [6]. An example of a 2% agarose gel displaying the results for 16 unknown horn samples can be seen in Fig 2. Only white rhino (44 out of 50) and black rhino (6 out of 50) horns were identified. The 10 remaining samples failed amplification in the multiplex.

## 4. Discussion

This work presents the first fast and accurate presumptive species identification test and casework data developed for one of the highest profile groups of animals involved in illegal wildlife trafficking, the rhinos’. The multiplex PCR assay described here significantly reduces the material and time costs of previous rhino horn species identification methods, whilst still providing a robust and accurate presumptive species identification for rhino horn. It is imperative that laboratories in Asian countries, where demand for these illegal products is currently highest, perform species identification testing to contribute to enforcement actions around horn traficking crimes and to monitor market trends [6, 7]. However, these laboratories may have limited capacity to produce timley and robust identifications. Currently, validated testing relies on Sanger sequencing [6] which may not be feasible for a number of reasons including a lack of labor, funding, infrastructure and/or expertise. This is an urgent concern for Vietnam, because the time required for transfer of material to the laboratory, DNA extraction and PCR amplification, and sequencing (which often takes weeks at IEBR) can hinder the chance of a successful prosecution. Our multiplex PCR assay was successfully implemented and field-tested at IEBR, by their own laboratory staff, and can now be used to facilitate timely and robust presumptive identifications for rhino horn trafficking investigations in Vietnam, allowing rapid and reliable information to flow back to enforcement agencies. Addtionally, it allows for testing of horns, to ensure animal disease regulations are met prior to sending samples to better equiped or specialised laboratories, such as the South African RhODIS laboratory [10].

The possibility of rapid turnaround has been achieved through the use of species-specific primers, designed to amplify different length regions within cyt b which are unique to the different rhino species. All primers are incorporated into a single multiplex PCR. The multiplex PCR protocol presented here can provide a ‘presumptive’ species identification within 24 hours, and can be confirmed with a more time consuming ‘definitive’ sequence based species identification method at a later stage if/when required (such as [6]). The ability to perform a species identification in a single reaction also reduces the risk of contamination (from handling), which may be an issue for laboratories that may not have the resources to enforce strict forensic standards.

**Fig 3:**
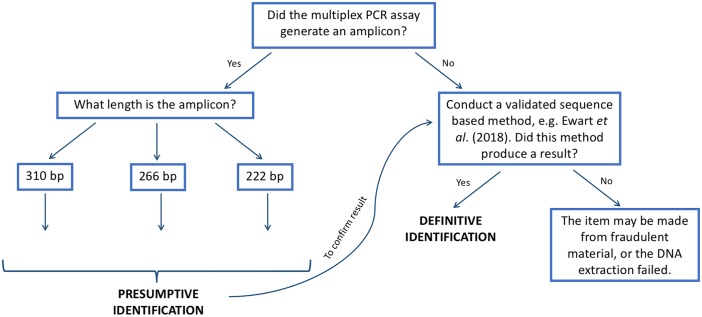
Identification key to interpret the results of the multiplex PCR assay.

All of the known rhino samples in developmental stage of the assay, and a significant proportion of the unknown horns (83%) in the field test were successfully identified using the multiplex PCR assay (S1 Table). Only white and black rhino horns were identified in the field test, consistent with rhino poaching trends [4, 15]. No Indian rhino horns were identified in the field test; however, all of the Indian rhino samples trialed in the development stage were successfully identified (4 out of 4 of the samples). We are therefore confident that the multiplex PCR assay can reliably identify Indian rhino horns, and that no Indian rhino horns were present in the seizure samples tested at IEBR. Fig 3 shows our recommendation for how the multiplex PCR assay should be interpreted for a range of different rhino horn identification scenarios.

It should be noted that there are a few caveats to this presumptive test. Firstly, the assay was limited to identify three of the five rhino species (black rhino, white rhino and Indian rhino). However, it is highly unlikely that Javan rhino or Sumatran rhino horns will be encountered in the rhino horn market as there are fewer than 100 and 300 individuals left respectively [16, 17]. Additionally, both of these species could be tested via a sequence based method, hence false negatives or misidentification as fraudulent horn product will be avoided (as in Fig 3). Secondly, the assay was designed to exclude non-target species, including Javan rhino, Sumatran rhino, water buffalo (potential false positives) and human (to account for possible human contamination). However, high quality and/or high concentration DNA samples were not available for Javan rhino, Sumatran rhino and water buffalo, which may compromise the specificity testing involving these species. Thirdly, 10 of the horn samples when field testing the multiplex PCR assay at IEBR did not produce a result. While it is possible that these samples were fraudulent horns (e.g. plastic), it is more likely the extraction and/or multiplex PCR assay failed for these samples due to poor quality and/or low template DNA (DNA quantification instruments were not available to investigate this possibility). Modifications to the DNA extraction protocols during the field testing at IEBR had to be made due to the equipment and infrastructure available. Different subsampling and extraction methods were not compared in formal experiments, however drilling and homogenizing the samples shortened the time needed to adequately lyse the horn samples. Optimizing the DNA extraction step at IEBR or using well established methods (for example, [8]) may have increased the species identification success of the multiplex PCR assay. Further work to optimize a more rapid and cheaper extraction method that could be implemented when restricted budgets are a concern, would be useful as the DNA extraction step is the most time-consuming and expensive part of the multiplex PCR assay protocol.

## 5. Conclusion

We are currently amidst a rhino poaching crisis, in which over 1000 rhinos are being poached each year [4, 15]. The multiplex PCR assay presented here provides an effective means to generate presumptive species identifications for rhino horn from commonly traded species within 24 hours of samples arriving at a laboratory. This assay will increase the speed and reduce the material and time costs of the current species identification techniques that rely on sequencing and sequence analysis by allowing for more efficient triaging of suspected horn products for further downstream analysis and potential enforcement. Increasing the capacity to conduct rhino horn investigations, particularly for ‘front-line’ laboratories such as IEBR, is a vital component to combat the trade [4, 18]. It is important to consistently identify the species of seized horn products, not only to assist in securing a conviction, but also to monitor the market trends of rhino horn trafficking in range state and destination countries.

It is hoped that the implementation of this new protocol at IEBR, and other laboratories in similar situations that also require rapid rhino horn identifications, will not only improve the enforcement of rhino horn trafficking crimes, but also serve as a model in which future ‘rapid species identification tests’ can be trialed.

## Supporting information

S1 Table Sample details and multiplex PCR species ID assay trial results during the developmental stage, and the subsequent field test results at IEBR. (DOCX)
