# Correction: A rapid multiplex PCR assay for presumptive species identification of rhinoceros horns and its implementation in Vietnam

**DOI:** 10.1371/journal.pone.0202433

**Published:** 2018-08-10

**Authors:** Kyle M. Ewart, Greta J. Frankham, Ross McEwing, Dang Tat The, Carolyn J. Hogg, Claire Wade, Nathan Lo, Rebecca N. Johnson

There are errors in Table 1. The amplicon lengths for primers RID_FWD/RID_REV and Mac_FWD/Mac_REV do not include primer regions. Please see the corrected Table 1 here.

**Table 1:** Cytochrome b markers and their corresponding primers.

Genetic marker for:	Primer name:	Primer sequence (5’–3’):	Annealing temperature (°C):	Amplicon length* (bp):	Reference:
*Diceros bicornis* (black rhino)	Rh_BR_FWD (forward)	AATCTGCCTAATCCTACAAATC	60	222	This study
	Rh_BR_REV (reverse)	GGTTTCTAGGAAGGTGTAGG			
*Ceratotherium simum* (white rhino)	Rh_WR_FWD (forward)	CCACTCATTCATCGATCTGC	60	266	This study
	Rh_WR_REV (reverse)	TAATAGATACCGCGTCCTAC			
*Rhinoceros unicornis* (Indian rhino)	Rh_IR_FWD (forward)	TCTCACCCACTAGTTAAAATCA	60	310	This study
	Rh_IR_REV (reverse)	AGGAAGGTGTAAGATCCATAG			
All rhino species	RID_FWD (forward)	AACATCCGTAAATCYCACCCA	55	274	[6]
	RID_REV (reverse)	GGCAGATRAARAATATGGATGCT			
All rhino species	Mac_FWD (forward	CAYTATACACCAGACACAACAAC	55	226	This study
	Mac_REV (reverse)	TGAAYGCDGTGGCTATTAGRG			

*Amplicon lengths are inclusive of primer regions.

## References

[1] Ewart KM, Frankham GJ, McEwing R, The DT, Hogg CJ, Wade C, et al (2018) A rapid multiplex PCR assay for presumptive species identification of rhinoceros horns and its implementation in Vietnam. PLoS ONE 13(6): e0198565 10.1371/journal.pone.0198565 29902212 PMC6002117

