# Supplementary material for: A rapid multiplex PCR assay for presumptive species identification of rhinoceros horns and its implementation in Vietnam
Source: PLoS One. 2018 Jun 14;13(6):e0198565. doi: 10.1371/journal.pone.0198565 (PMC6002117; doi:10.1371/journal.pone.0198565)
Supplement: S1 Table — (DOCX) [file pone.0198565.s001.docx]

**A Rapid Multiplex PCR Assay for Presumptive Species Identification of Rhinoceros Horns and its Implementation in Vietnam**

**Supporting Information**

Kyle M. Ewart, Greta J. Frankham, Ross McEwing, Dang Tat The, Carolyn J. Hogg, Claire Wade, Nathan Lo, Rebecca N. Johnson

**S1 Table. Sample details and multiplex PCR species ID assay trial results during the developmental stage, and the subsequent field test results at IEBR.**

| **Sample:** | **Species:** | **Sample origin:** | **Sample type:** | **Sample use:** | **Multiplex PCR assay result:** |
| --- | --- | --- | --- | --- | --- |
| M.47175.001 | *Diceros bicornis* (black rhino) | Seized specimen | Horn | Development stage trial | Black rhino |
| M.47195.001 | *Diceros bicornis* (black rhino) | Zoo collection | Horn | Development stage trial | Black rhino |
| M.47204.001 | *Diceros bicornis* (black rhino) | Zoo collection | Blood | Development stage trial | Black rhino |
| M.47156.001 | *Ceratotherium simum* (white rhino) | Zoo collection | Horn | Development stage trial | White rhino |
| M.47183.001 | *Ceratotherium simum* (white rhino) | Zoo collection | Horn | Development stage trial | White rhino |
| M.47187.001 | *Ceratotherium simum* (white rhino) | Zoo collection | Hair | Development stage trial | White rhino |
| M.39432.005 | *Rhinoceros unicornis* (Indian rhino) | Museum collections | Tissue | Development stage trial | Indian rhino |
| M.43472.001 | *Rhinoceros unicornis* (Indian rhino) | Museum collections | Horn | Development stage trial | Indian rhino |
| M.47176.001 | *Rhinoceros unicornis* (Indian rhino) | Seized specimen | Horn | Development stage trial | Indian rhino |
| M.39432.003(a) | *Rhinoceros unicornis* (Indian rhino) | Museum collections | Skull | Development stage trial | Indian rhino |
| WGM105_02 | Unknown | IEBR seized specimen | Horn | Field test | White rhino |
| WGM105_03 | Unknown | IEBR seized specimen | Horn | Field test | White rhino |
| WGM105_04 | Unknown | IEBR seized specimen | Horn | Field test | Black rhino |
| WGM105_05 | Unknown | IEBR seized specimen | Horn | Field test | White rhino |
| WGM105_06 | Unknown | IEBR seized specimen | Horn | Field test | White rhino |
| WGM105_07 | Unknown | IEBR seized specimen | Horn | Field test | White rhino |
| WGM105_08 | Unknown | IEBR seized specimen | Horn | Field test | White rhino |
| WGM105_09 | Unknown | IEBR seized specimen | Horn | Field test | White rhino |
| WGM105_11 | Unknown | IEBR seized specimen | Horn | Field test | White rhino |
| WGM105_12 | Unknown | IEBR seized specimen | Horn | Field test | White rhino |
| WGM105_13 | Unknown | IEBR seized specimen | Horn | Field test | White rhino |
| WGM105_14 | Unknown | IEBR seized specimen | Horn | Field test | Black rhino |
| WGM105_15 | Unknown | IEBR seized specimen | Horn | Field test | White rhino |
| WGM105_17 | Unknown | IEBR seized specimen | Horn | Field test | White rhino |
| WGM105_18 | Unknown | IEBR seized specimen | Horn | Field test | White rhino |
| WGM105_19 | Unknown | IEBR seized specimen | Horn | Field test | White rhino |
| WGM105_20 | Unknown | IEBR seized specimen | Horn | Field test | Fail |
| WGM105_22 | Unknown | IEBR seized specimen | Horn | Field test | Black rhino |
| WGM105_23 | Unknown | IEBR seized specimen | Horn | Field test | White rhino |
| WGM105_24 | Unknown | IEBR seized specimen | Horn | Field test | White rhino |
| WGM105_26 | Unknown | IEBR seized specimen | Horn | Field test | Fail |
| WGM105_27 | Unknown | IEBR seized specimen | Horn | Field test | White rhino |
| WGM105_28 | Unknown | IEBR seized specimen | Horn | Field test | White rhino |
| WGM105_29 | Unknown | IEBR seized specimen | Horn | Field test | White rhino |
| WGM105_31 | Unknown | IEBR seized specimen | Horn | Field test | White rhino |
| WGM105_32 | Unknown | IEBR seized specimen | Horn | Field test | Fail |
| WGM105_33 | Unknown | IEBR seized specimen | Horn | Field test | Fail |
| WGM105_34 | Unknown | IEBR seized specimen | Horn | Field test | Fail |
| WGM105_35 | Unknown | IEBR seized specimen | Horn | Field test | Fail |
| WGM105_36 | Unknown | IEBR seized specimen | Horn | Field test | White rhino |
| WGM105_37 | Unknown | IEBR seized specimen | Horn | Field test | White rhino |
| WGM105_38 | Unknown | IEBR seized specimen | Horn | Field test | White rhino |
| WGM105_39 | Unknown | IEBR seized specimen | Horn | Field test | Black rhino |
| WGM105_40 | Unknown | IEBR seized specimen | Horn | Field test | Fail |
| TG01 | Unknown | IEBR seized specimen | Horn | Field test | White rhino |
| TG02 | Unknown | IEBR seized specimen | Horn | Field test | White rhino |
| TG03 | Unknown | IEBR seized specimen | Horn | Field test | Black rhino |
| TG04 | Unknown | IEBR seized specimen | Horn | Field test | White rhino |
| TG05 | Unknown | IEBR seized specimen | Horn | Field test | White rhino |
| TG06 | Unknown | IEBR seized specimen | Horn | Field test | White rhino |
| TG07 | Unknown | IEBR seized specimen | Horn | Field test | White rhino |
| TG10 | Unknown | IEBR seized specimen | Horn | Field test | White rhino |
| TG11 | Unknown | IEBR seized specimen | Horn | Field test | White rhino |
| TG12 | Unknown | IEBR seized specimen | Horn | Field test | Black rhino |
| TG13 | Unknown | IEBR seized specimen | Horn | Field test | White rhino |
| TG14 | Unknown | IEBR seized specimen | Horn | Field test | White rhino |
| TG16 | Unknown | IEBR seized specimen | Horn | Field test | White rhino |
| TG17 | Unknown | IEBR seized specimen | Horn | Field test | White rhino |
| TG18 | Unknown | IEBR seized specimen | Horn | Field test | White rhino |
| TG19 | Unknown | IEBR seized specimen | Horn | Field test | White rhino |
| TG20 | Unknown | IEBR seized specimen | Horn | Field test | White rhino |
| TG21 | Unknown | IEBR seized specimen | Horn | Field test | Fail |
| TG22 | Unknown | IEBR seized specimen | Horn | Field test | Fail |
| TG23 | Unknown | IEBR seized specimen | Horn | Field test | White rhino |
| TG25 | Unknown | IEBR seized specimen | Horn | Field test | White rhino |
| TG26 | Unknown | IEBR seized specimen | Horn | Field test | White rhino |
| TG27 | Unknown | IEBR seized specimen | Horn | Field test | Fail |
| TG28 | Unknown | IEBR seized specimen | Horn | Field test | White rhino |
| TG30 | Unknown | IEBR seized specimen | Horn | Field test | White rhino |
| TG31 | Unknown | IEBR seized specimen | Horn | Field test | White rhino |
